# Supplementary material for: P18: Novel Anticancer Peptide from Induced Tumor-Suppressing Cells Targeting Breast Cancer and Bone Metastasis
Source: Cancers (Basel). 2024 Jun 15;16(12):2230. doi: 10.3390/cancers16122230 (PMC11202002; doi:10.3390/cancers16122230)
Supplement: Supplementary file 1 [file cancers-16-02230-s001.zip › cancers-3013221-figures and tables.pdf]

Suppl. Table S1. Sequence and characterization of peptides P11 to P22.

ID	amino acid sequence	host gene symbol	MW (Da)	isoelectric point	charge (pH7)	average hydrophilicity	hydrophilic residues (%)	iDCP score
P11	FEDENFILK	Ppia	1154	3.77	-2	0.40	56%	0.23
P12	VNFTVDQIR	Eef2	1091	6.85	0	-0.14	44%	0.26
P13	NDLAVVDVR	Ncl	1000	3.87	-1	0.27	44%	0.14
P14	EEAESTLQSFR	Vim	1296	3.95	-2	0.69	64%	0.13
P15	IDTIEIITDR	Hnrn	1188	3.64	-2	0.40	40%	0.32
P16	YEWDVAEAR	Eef2	1138	3.77	-2	0.42	44%	0.22
P17	LGDLYEEEMR	Vim	1254	3.58	-3	0.78	50%	0.23
P18	TDYMVGSYGPR	Arhgdia	1245	6.70	0	-0.14	27%	0.26
P19	DQVANSAFVER	Hsp90aa1	1235	4.09	-1	0.29	55%	0.23
P20	QITLNDLPVGR	Prdx4	1225	6.85	0	-0.08	36%	0.15
P21	FEELNADLFR	Hspa8	1253	3.77	-2	0.31	50%	0.12
P22	IEVIEIMTDR	Hnrnpa1	1218	3.77	-2	0.34	40%	0.16

Suppl. Table S2. MTT-based relative viability of 2 breast cancer cell lines.

	**cn**	**P11**	**P12**	**P13**	**P14**	**P15**
MDA-MB-231	1.00	0.96	0.94	0.98	0.88	0.93
MDA-MB-436	1.00	0.95	0.92	0.95	0.84	0.95

	**P16**	**P17**	**P18**	**P19**	**P20**	**P21**	**P22**
MDA-MB-231	0.93	1.10	0.86	1.09	0.87	0.89	0.86
MDA-MB-436	0.96	0.99	0.82	0.96	0.86	0.92	0.88

Suppl. Table S3. Hydrogen-bonding interaction in ARHGDIA-Cdc42 complex.

ARHGDIA Receptor Residue	Cdc42 Ligand Residue	Interaction Constituents	Distance (Å)
A: TYR27	C: ASP63	A:TYR27:OH-C:ASP63:OD2	2.16
A: TYR51	C: ASP65	A:TYR27:OH-C:ASP65:OD2	2.21
A: CYS79	C: ASP122	A:TYR27:N-C:ASP122:OD1	3.68
A: CYS79	C: SER124	A:TYR27:N-C:SER124:OG	3.56
A: ARG111	C: ASP118	A:TYR27:NH2-C:ASP118:OD2	2.43
A: LYS113	C: THR125	A:TYR27:NZ-C:THR125:O	2.77
A: SER115	C: LYS128	A:TYR27:OG-C:LYS128:O	3.48
A: SER124	C: ASP63	A:TYR27:OG-C:ASP63:OD1	3.55
A: SER124	C: ASP63	A:TYR27:OG-C:ASP63:OD2	3.65
A: ARG152	C: GLU91	A:TYR27:NH1-C:GLU91:OE2	3.65
A: GLU157	C: SER86	A:TYR27:O-C:SER86:OG	3.54
A: PHE158	C: LYS128	A:TYR27:O-C: LYS128:NZ	2.73
A: GLU87	C: LYS131	A:TYR27:OE2-C:LYS131:NZ	2.10
A: TYR156	C: ASN132	A:TYR27:O-C:ASN132:ND2	3.55

Suppl. Table S4. Hydrogen-bonding interaction in P18-Cdc42 complex.

P18 Ligand Residue	Cdc42 Receptor Residue	Interaction Constituents	Distance (Å)
C: ASP143	A: LYS128	A:LYS128:NZ-C:ASP143:O	2.73
C: ASP143	A: LEU129	A:LEU129:N-C:ASP143:OD2	3.15
C: TYR144	A: SER88	A:SER88:OG-C:TYR144:O	3.75
C: TYR144	A: SER86	A:SER86:OG-C:TYR144:O	3.09
C: THR142	A: THR125	A:THR125:OG1-C:THR142:N	3.72
C: VAL146	A: SER88	A:SER88:OG-C:VAL146:N	3.03
C: ARG152	A: ASP65	A:ASP65:OD2-C:ARG152:NH2	3.37

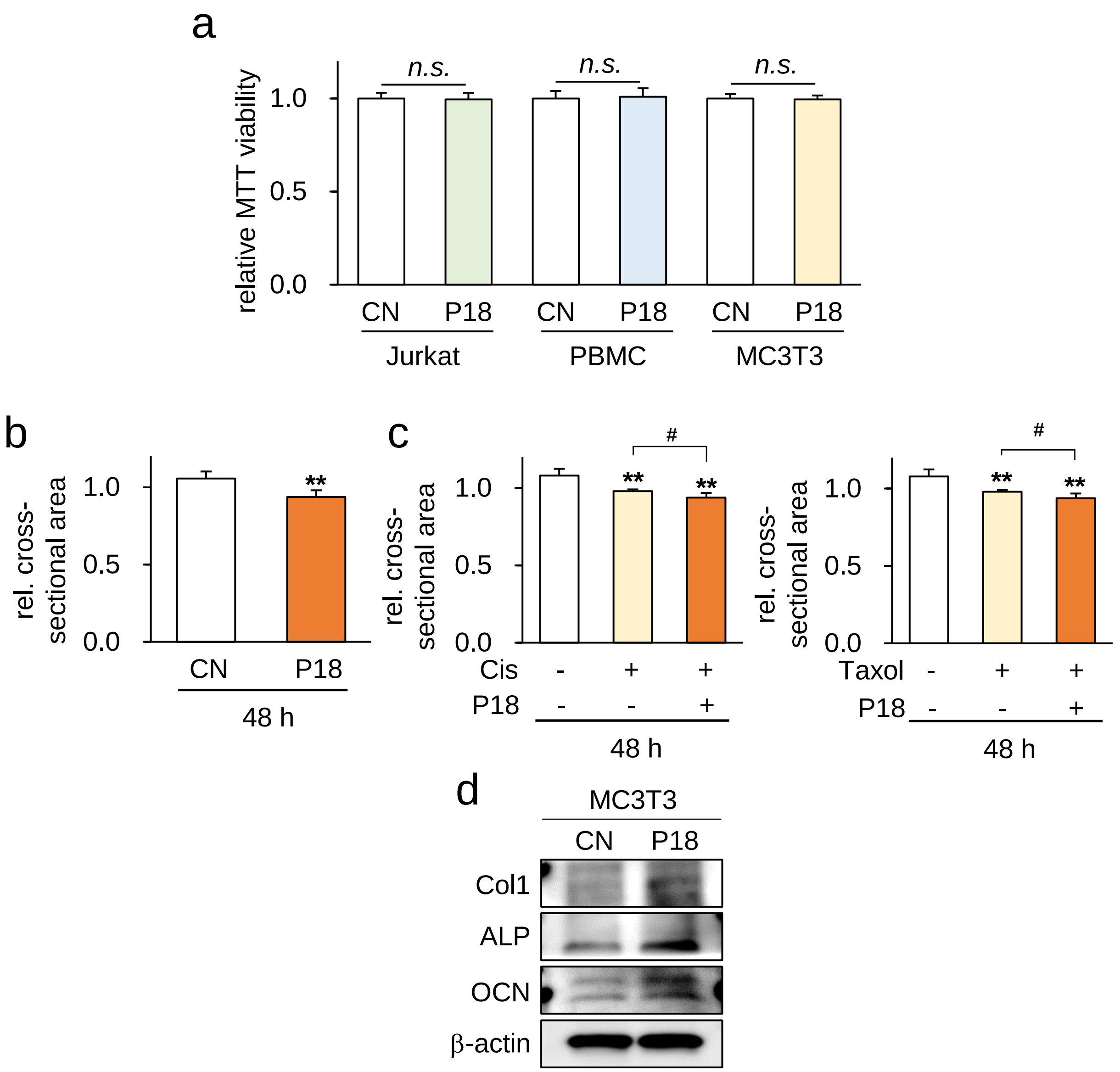

Suppl. Figure S1

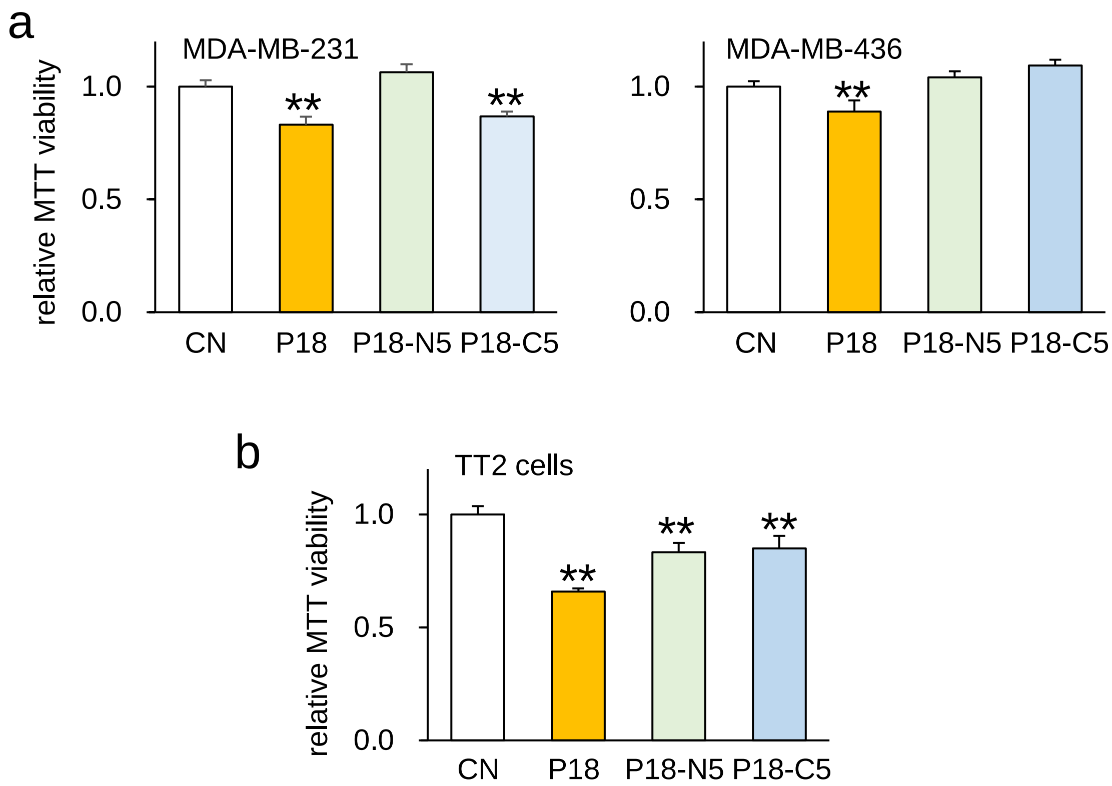

Suppl. Figure S2

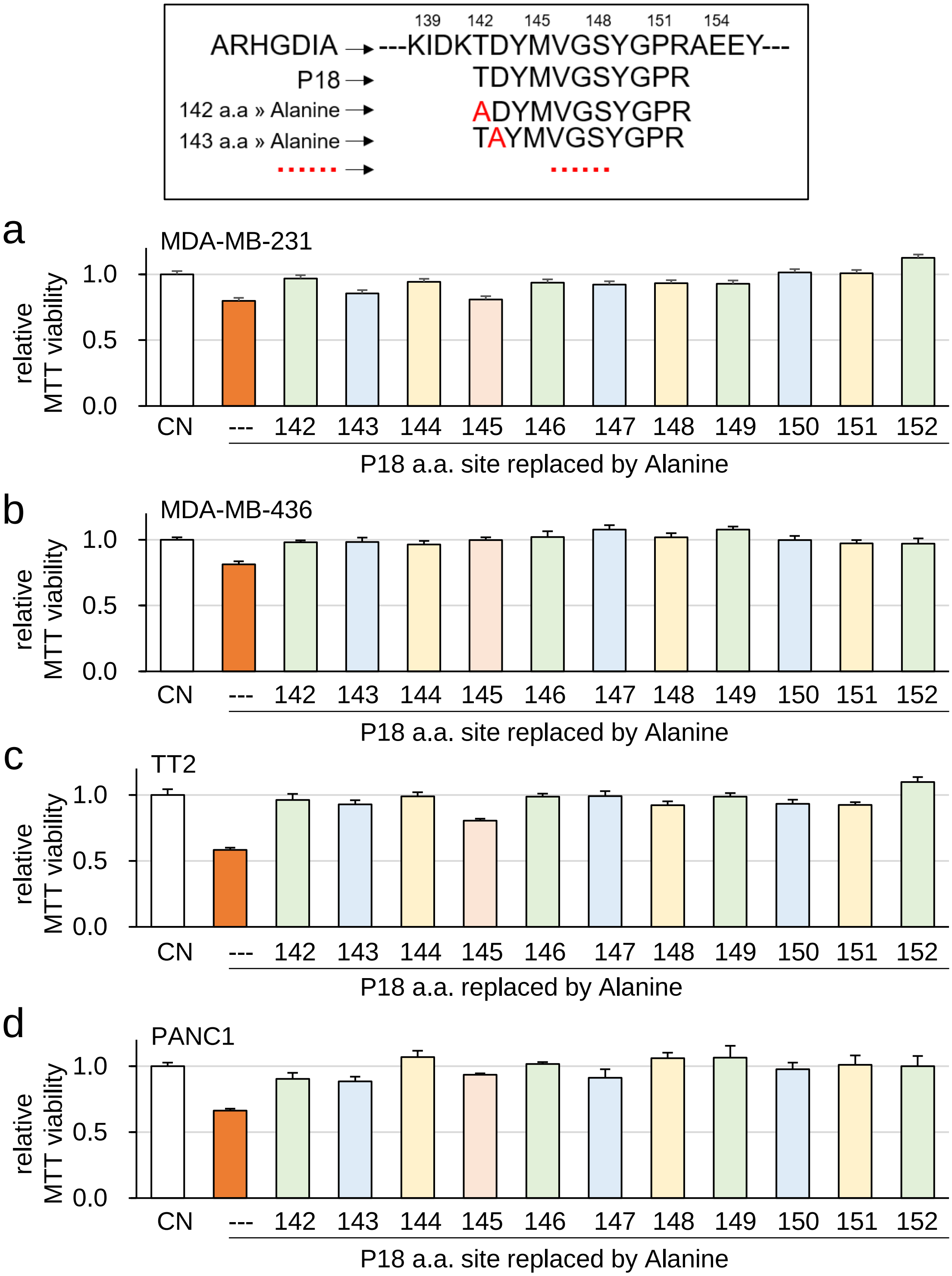

Suppl. Figure S3

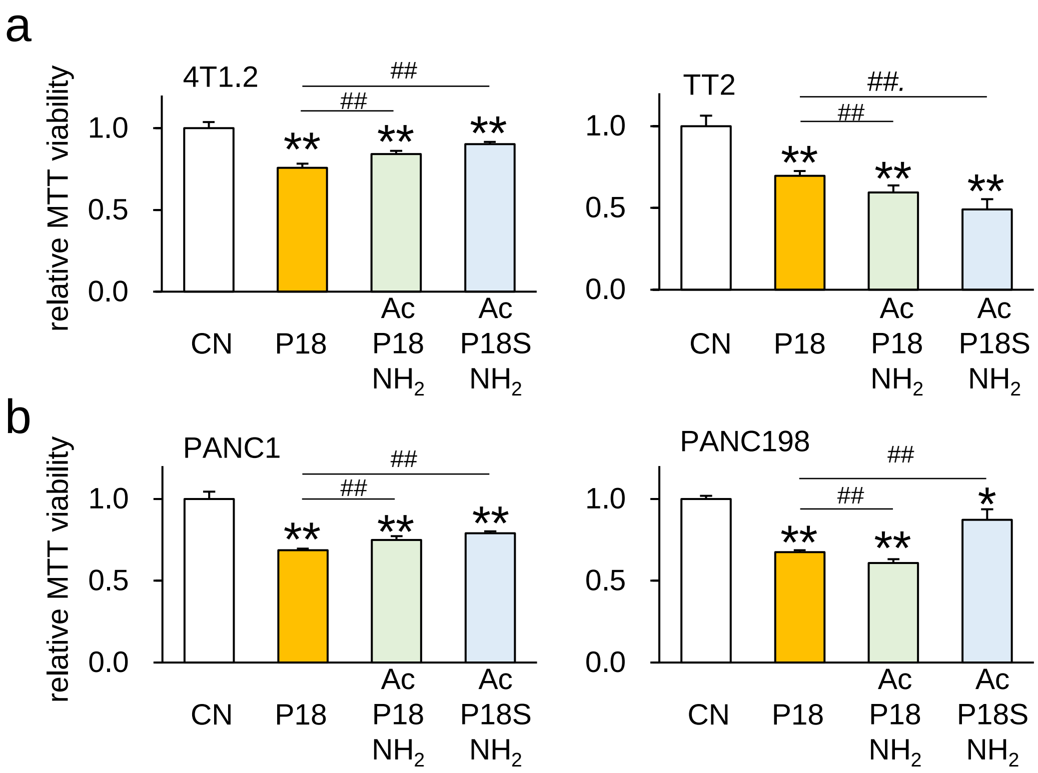

Suppl. Figure S4

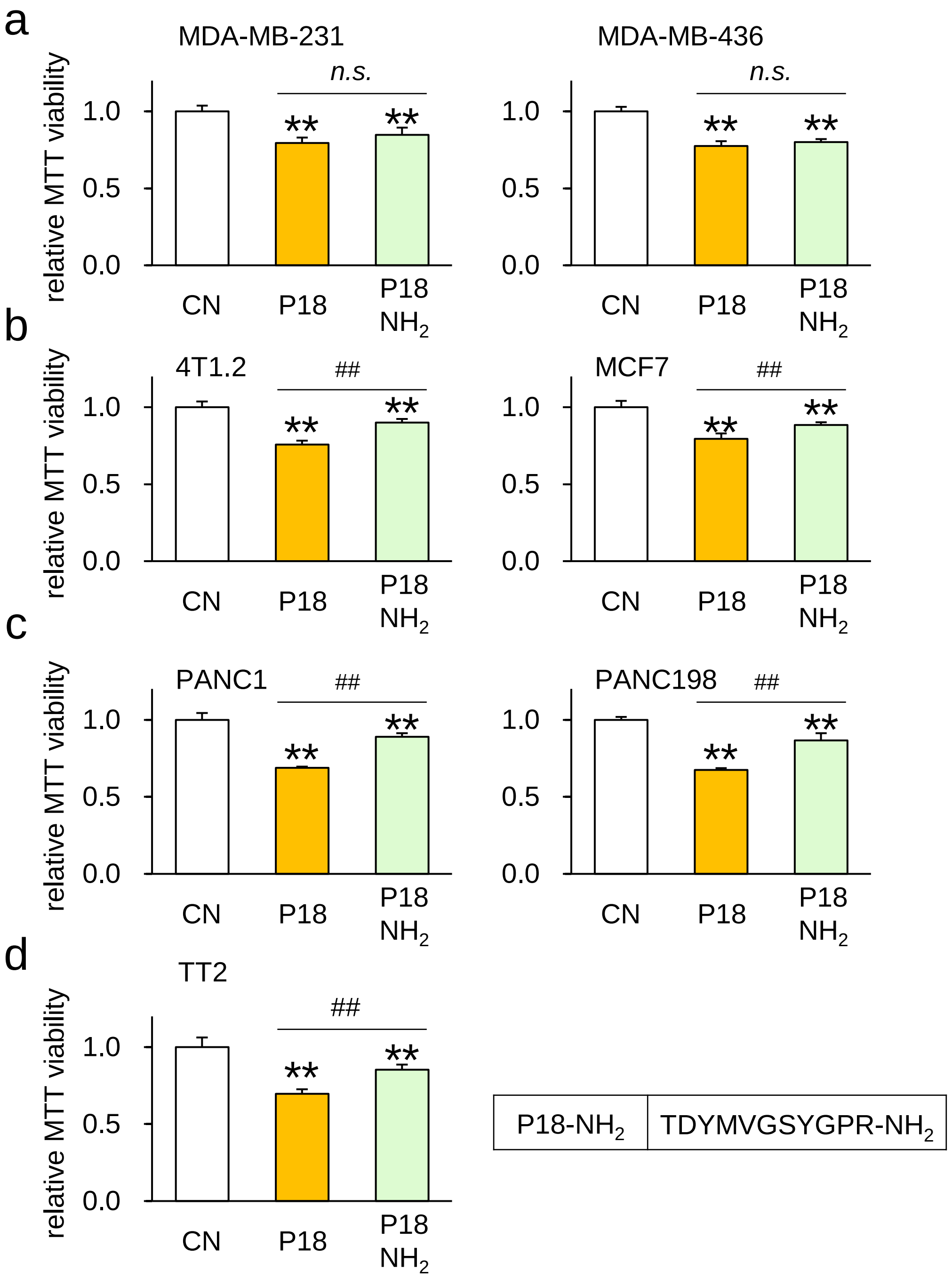

Suppl. Figure S5
