# Supplementary material for: P18: Novel Anticancer Peptide from Induced Tumor-Suppressing Cells Targeting Breast Cancer and Bone Metastasis
Source: Cancers (Basel). 2024 Jun 15;16(12):2230. doi: 10.3390/cancers16122230 (PMC11202002; doi:10.3390/cancers16122230)
Supplement: Supplementary file 1 [file cancers-16-02230-s001.zip › cancers-3013221-File S1. original-images.pdf]

Original gels (images used in the manuscript and supplementary information)

Figure 4

Fig. 4b

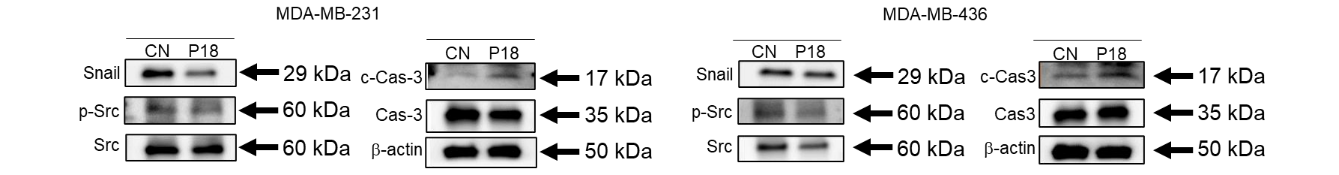

Figure 5

Fig. 5d

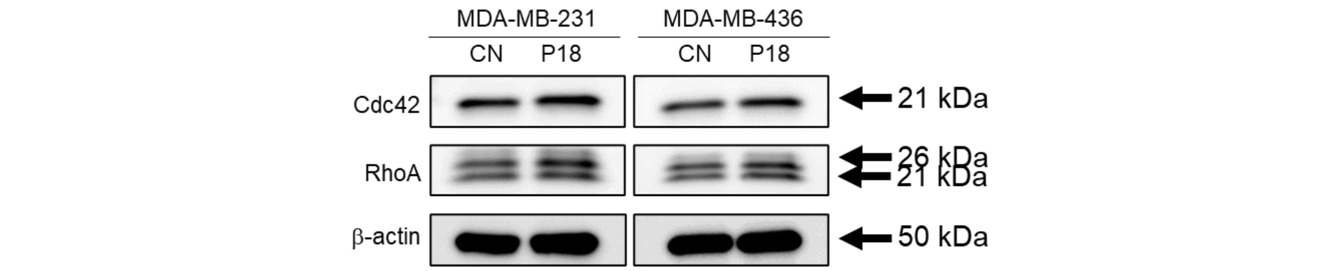

Figure 8

Fig. 8a

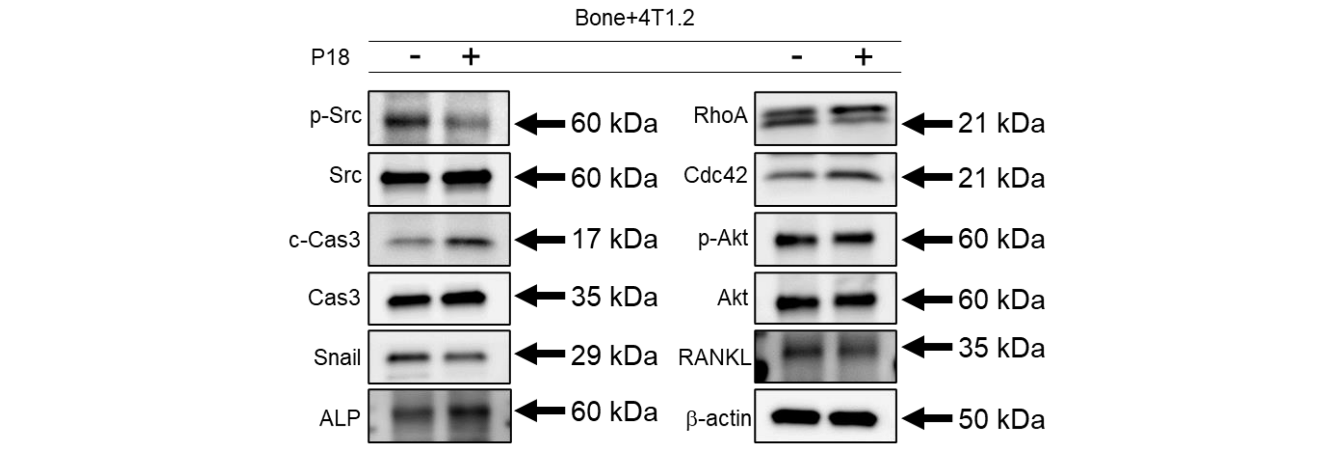

Uncropped gels (images we can see the edges of the membrane and markers if possible)

Figure 4

Fig. 4b

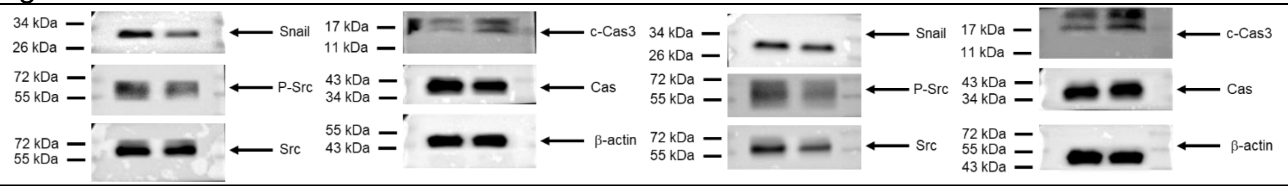

Figure 5

Fig. 5d

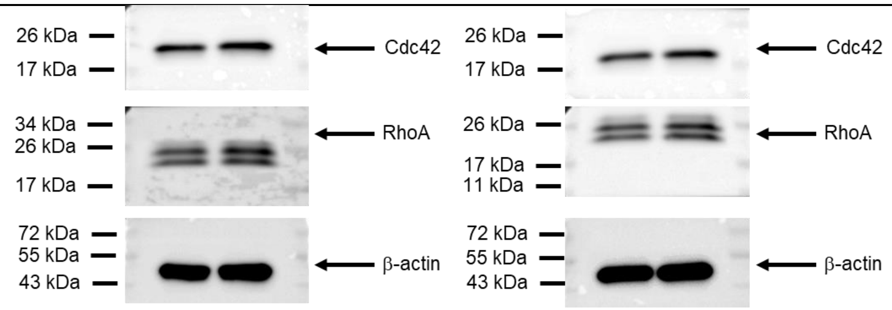

Figure 8

Fig. 8a

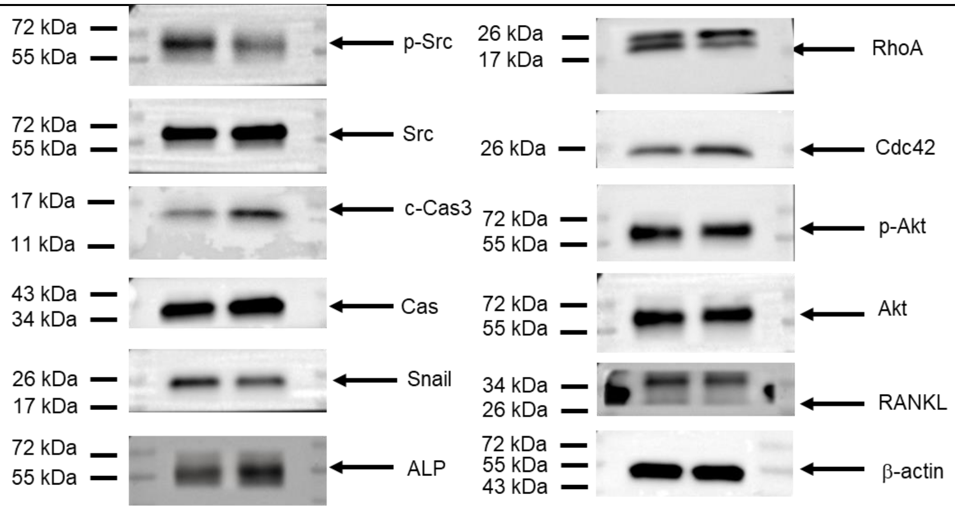

Untouched gels (Only emphasize which bands used in the paper)
Figure 4

Fig. 4b

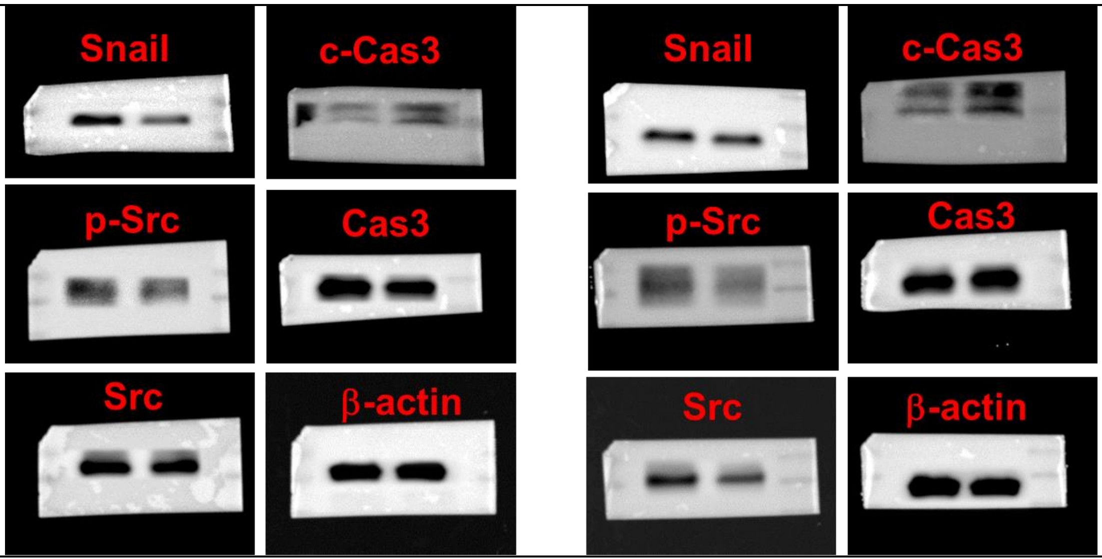

Figure 5

Fig. 5d

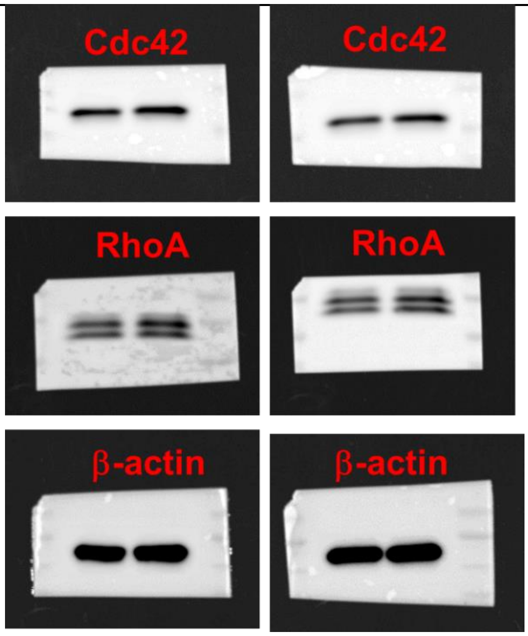

Figure 8

Fig. 8a

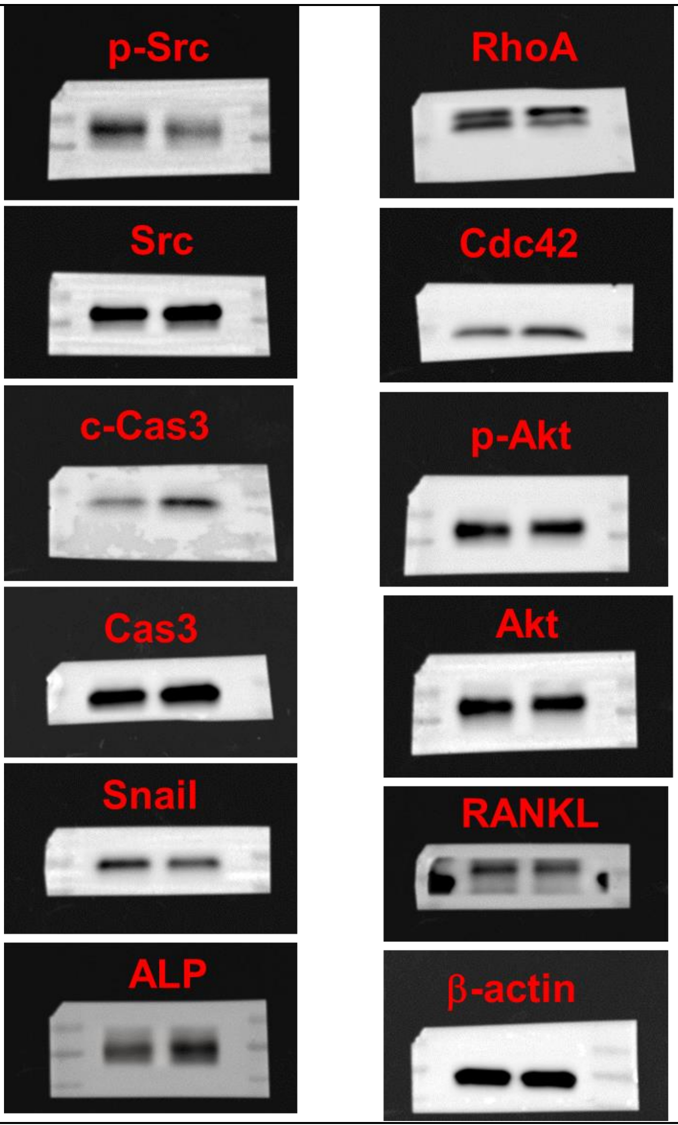
p-Src
Src
c-Cas3
Cas3
Snail
ALP
RhoA
Cdc42
p-Akt
Akt
RANKL
β-actin
